# The thick waxy coat of mycobacteria, a protective layer against antibiotics and the host's immune system

**DOI:** 10.1042/BCJ20200194

**Published:** 2020-05-29

**Authors:** Sarah M. Batt, David E. Minnikin, Gurdyal S. Besra

**Affiliations:** School of Biosciences, Institute of Microbiology and Infection, University of Birmingham, Edgbaston, B15 2TT Birmingham, U.K.

**Keywords:** biosynthesis, cell wall, lipids, mycobacterium tuberculosis, mycolic acids

## Abstract

Tuberculosis, caused by the pathogenic bacterium *Mycobacterium tuberculosis* (*Mtb*), is the leading cause of death from an infectious disease, with a mortality rate of over a million people per year. This pathogen's remarkable resilience and infectivity is largely due to its unique waxy cell envelope, 40% of which comprises complex lipids. Therefore, an understanding of the structure and function of the cell wall lipids is of huge indirect clinical significance. This review provides a synopsis of the cell envelope and the major lipids contained within, including structure, biosynthesis and roles in pathogenesis.

## Introduction

*Mycobacterium tuberculosis* (*Mtb*), the causative agent of tuberculosis, was responsible for 1.5 million fatalities worldwide in 2018 [1]. Added to this are the growing concerns of a surge in strains resistant to front-line drugs (multidrug resistant; MDR) and those additionally resistant to second-line drugs (extremely drug resistant; XDR) and all known drugs (totally drug resistant; TDR) [2]. The distinctive cell envelope of *Mtb* is robust and waxy [3], thanks to the rich variety of lipids present, which bestow on this pathogen resilience [4], a hydrophobic barrier to antibiotic entry [5,6] and key virulence factors [7–10]. In view of these facts and since many of the existing antibiotics available for tuberculosis target the synthesis and transport of these complex lipids, an understanding of lipid structures and synthesis in *Mtb* is a major goal of current research, with promise of novel effective drug targets.

**Figure 1.**
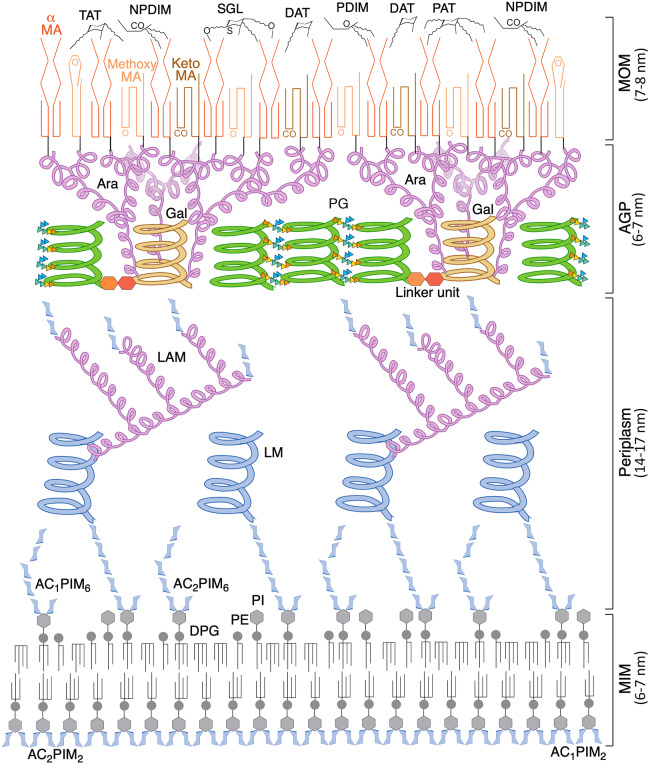
Structure of the cell envelope of *Mtb*. In the MIM (mycobacterial inner membrane), the inner leaflet mostly consists of AC_1/2_PIM_2_ (tri-/tetra-acylated phosphatidyl-*myo*-inositol-dimannoside). The outer leaflet also contains PIMs, in the form of AC_1/2_PIM_6_ (tri-/tetra-acylated phosphatidyl-*myo*-inositol-hexamannoside), as well as the more conventional phospholipids of the inner membrane, DPG (diphosphatidylglycerol), PE (phosphatidylethanolamine) and PI (phosphatidylinositol). In the periplasm, LM (lipomannan) and LAM (lipoarabinomannan) extend outwards, from lipid anchors in the MIM, though additional anchorage in the outer membrane has been demonstrated, which is contentious; the mannose core helix and individual sugar molecules of the PIMs and mannose caps are coloured blue and the branched arabinan domain is pink. The glycan backbone of the PG (peptidoglycan), green, is represented by helices positioned perpendicular to the membrane, according to the scaffold model [281, 282]. Peptide cross-links between the glycan helices are shown as coloured triangles (orange = l-alanine, yellow = d-*iso*glutamine, green = meso-diaminopimelate and blue = d-alanine). The PG is connected to the Gal (galactan) domain, yellow, of the arabinogalactan (AG) via a linker unit. The branched Ara (arabinan) domain of the AG, pink, is esterified with MA (mycolic acids), comprising the inner leaflet of the MOM (mycobacterial outer membrane); the typical conformation adopted by each class of MA is represented. The outer leaflet of the MOM is characterised by DAT, TAT and PAT (di-/tri- and pentaacyl trehalose); PDIM and NPDIM (dimycocerosates of phthiocerols and phthiodiolones); and SGL (sulphated trehalose glycolipids) [23,283]. The diagram is roughly to scale using dimensions obtained from cryo-electron microscopy [13].

Neither fully Gram-negative nor Gram-positive, the envelope of mycobacteria (Figure 1) is proposed to adopt a remarkable dual membrane structure, with a specialised mycobacterial outer membrane or ‘MOM’, formed as an inner layer of mycolic acids (MAs) and an outer leaflet of free lipids [5,11–13]. While the free lipids are not attached and can be stripped from the envelope, the MA layer is covalently linked to the polysaccharide cell wall [5,11,14], a feature not seen in Gram-negative bacteria. This connection forms part of the mycoloylarabinogalactan–peptidoglycan complex (mAGP), consisting of branched arabinose domains esterified on the outer surface with MAs and linked to the peptidoglycan via the galactan helices of the arabinogalactan (AG) [15–18]. The periplasmic space of *Mtb* is sizeable, allowing for enzymatic reactions to take place and for the material to be processed and translocated to the MOM [13,19–21]. The inner membrane of *Mtb* was initially assumed to be conventional, though lipid studies have since revealed that the phospholipid composition is predominantly phosphatidyl-*myo*-inositol mannosides (PIMs). More than half is present as PIM_2_ (with two mannose sugars on the inositol moiety), which appears to be exclusively within the inner leaflet of the inner membrane; while PIM_6_ (six mannose residues) is thought to occupy the outer leaflet [22]. This unusual arrangement increases the content of fatty acid chains in the inner membrane as Ac_1_/Ac_2_PIM_2_ and Ac_1_/Ac_2_PIM_6_, each has three or four (Ac_1_ or Ac_2_) acyl chains. The subsequent tight packing of these chains could lead to a less fluid, more stable membrane with reduced permeability to drugs [22], coining a new term mycobacterial inner membrane or ‘MIM’ [23]. The lipoglycans, lipomannan (LM) and lipoarabinomannan (LAM) have lipid anchors based on PIMs and probably project into the periplasm from the MIM. Some models of the *Mtb* cell envelope depict a capsule as the outermost layer beyond the MOM, which consists mainly of polysaccharides and proteins, with only small quantities of lipids [24]. This has been observed using cryo-electron microscopy to have a depth of ∼30 nm, but only in cultures grown under optimal conditions [25].

## Mycolic acids

**Figure 2.**
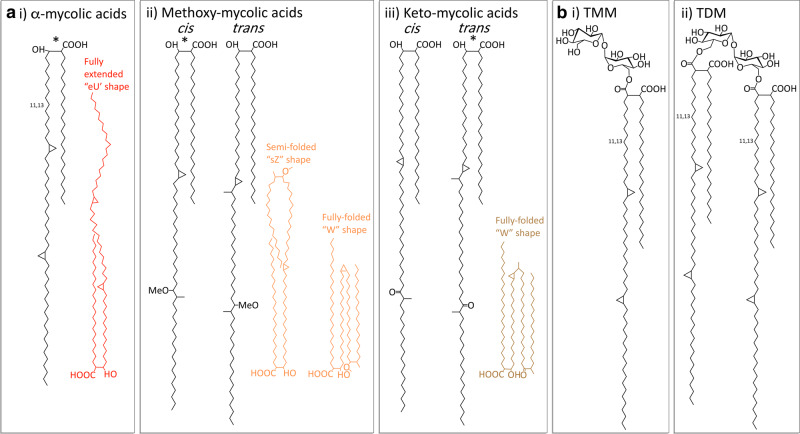
Structure of the major mycolic acid (MA) components from *Mtb*. (**a**) MA structures and the typical conformations adopted, (**i**) α-MAs, (**ii**) Methoxy-MAs and (**iii**) Keto-MAs. The main components are denoted with a *. (**b**) (**i**) TMM (trehalose monomycolates) and (**ii**) TDM (trehalose dimycolates) [284].

MAs are an abundant feature of the cell envelope of mycobacteria, representing 30% of the dry weight [26]; they provide a hydrophobic permeability barrier to antibiotics [6,27,28] and are essential for viability [29,30] and pathogenesis [31–35]. Characterised as long-chain α-alkyl-β-hydroxy fatty acids (70–90 carbons), most are covalently bound to the AG, forming the inner leaflet of the MOM [5,36]. While two-thirds of the AG termini are esterified with MAs [5,36], some MAs are present as extractable lipids in the outer leaflet of the MOM, mainly linked to trehalose as trehalose dimycolates (TDM) and trehalose monomycolates (TMM), or as free mycolates (Figure 2b). While these are thought to be mostly intermediates in the attachment of the MA to the AG [37], there is evidence to suggest that TDM, the so-called ‘cord-factor’ and most highly expressed glycolipid in *Mtb*, also has dedicated roles in pathogenicity through interactions with the C-type lectin receptor Mincle on the macrophage, which prevents phagosome acidification and promotes granuloma formation [38–41]. Free mycolates are more abundant in latent phase cells and could also play a role in biofilm formation [42,43].

MA structure can be separated into two parts: the meromycolate moiety consists of a long-chain meroaldehyde (up to 56 carbons) and usually has two functional groups, whereas the α-branch is a shorter, saturated alkyl chain (24–26 carbons) [3]. There are three classes of MAs in *Mtb*: α-mycolates are the most abundant and are present with only *cis*-cyclopropane rings, while oxygenated methoxymycolates and ketomycolates both have subclasses with predominantly either *cis*- or *trans*-cyclopropane rings and an adjacent methyl branch [5,6] (Figure 2a).

The MA chains are packed tightly into parallel arrays perpendicular to the surface of the cell envelope, which may be stabilised by hydrogen bonds between the β-OH groups [5,6,36,44]. Within these arrays, the MAs fold into energetically favourable conformations, modulated by the *cis*-double bonds and the cyclopropane rings, which introduce kinks into the long chains [6]; stabilising hydrophilic interactions are also formed through the oxygen functions [45]. This enables the keto-MAs to adopt a ‘W-form’ conformation, in which four chains are packed together in parallel [45,46]. Though the α-MAs and methoxy-MAs can also form a ‘W’ shape, they are more flexible, particularly the α-MAs, adopting more open forms at higher temperatures (Figure 2a) [47]. This tight packing provides the MA monolayer with appropriate rigidity, leading to a hydrophobic permeability barrier, which could be regulated by the MAs assuming altered conformations depending on their structure and the ambient temperature [45]. Each mycobacterial species has a specific MA profile, suggesting that the different structural elements have an important biological role, both physiologically and in pathogenicity [48].

## Free lipids

**Figure 3.**
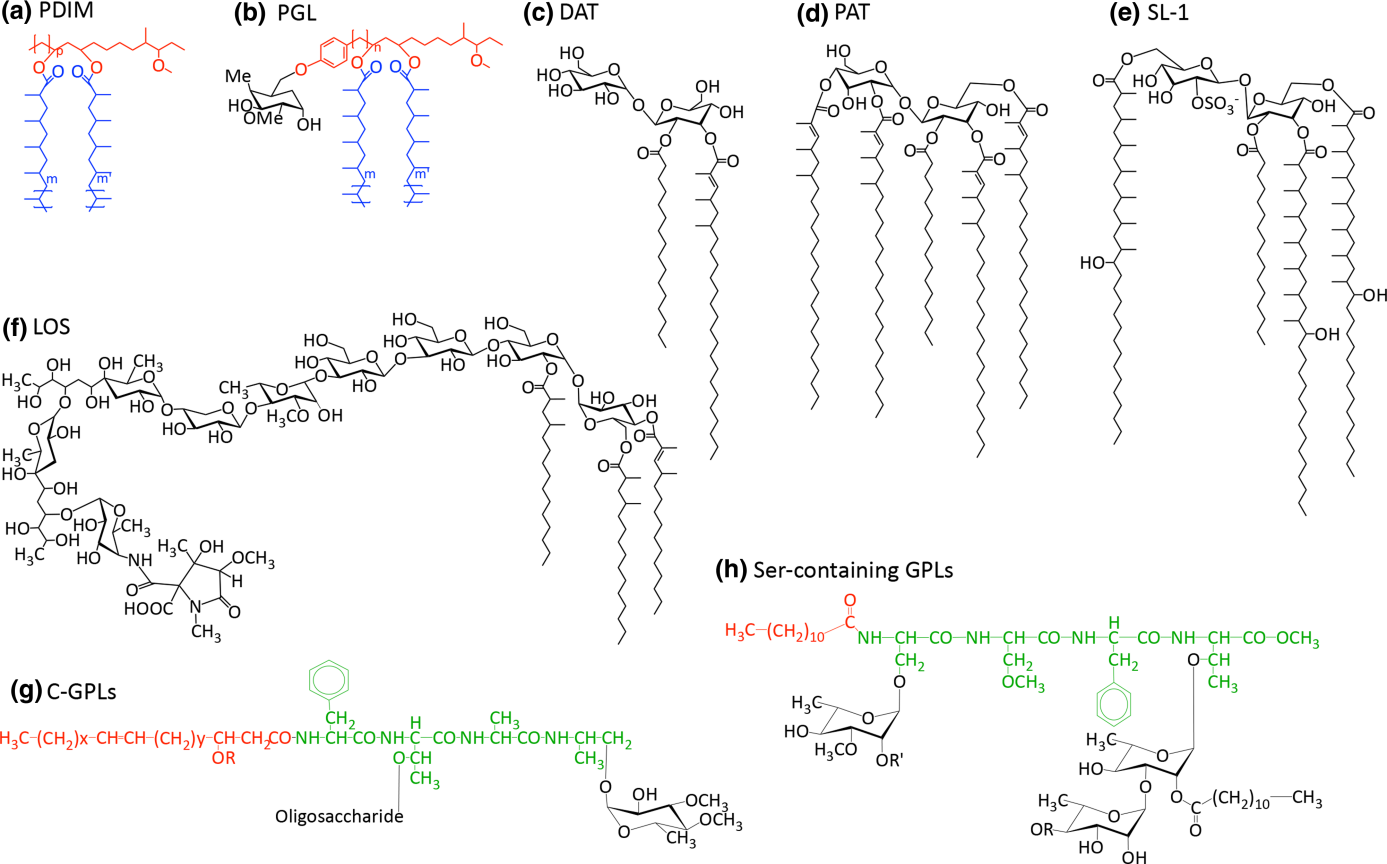
Free lipid structures. (**a**) PDIM (Phthiocerol dimycocerosate) and (**b**) PGL (phenolic glycolipids, general structure): black = saccharide; red = phenolphthiocerol/phthiocerol; blue = multimethyl-branched fatty acids (mycocerosic acids) [285], (**c**) DAT (diacyl trehaloses) [286], (**d**) PAT (pentaacyl trehaloses) [286], (**e**) Sulfolipid-1, SL-1 (sulphated trehalose glycolipid, SGL) [286] (**f**) Lipooligosaccharides (LOS) from *M. marinum* [142], (**g**) C-GPLs (general structure) [103] and (**h**) Ser-containing GPL from *M. xenopi*: red = lipid; green = peptide; black = saccharide [106].

Phthiocerol dimycocerosate (PDIMs) and phenolic glycolipids (PGLs) (Figure 3a,b) are large ( ∼90 carbons), complex, hydrophobic molecules found in the outer leaflet of the MOM [49,50]. The unusual lipid core consists of a long-chain β-diol (phthiocerol) esterified with two multimethyl branched long-chain fatty acids (mycocerosic acids) [49,51–53]. Whilst possessing a similar lipid core, PGLs differ through a phenolic residue that forms a link between this core and a chain of up to four saccharide units; the composition of the oligosaccharide is species-specific and confers antigenic properties [5]. The stereochemistry of the diol units of PDIMs and PGLs also vary by species; *M. marinum* and *M. ulecerans* both have *erythro* stereochemistry, while all other strains of mycobacteria have the *threo* configuration [54–56]. PDIMs are produced by all virulent strains of *Mtb* and have important roles in pathogenicity: forming an impermeable barrier on the outer surface of the cell [57,58], masking the cell from the host's immune system and lysing the phagosome in order to escape the macrophage and disseminate [59,60]. Interestingly, PDIMs give a negative advantage to growth *in vitro*, leading to their spontaneous loss, though they are never absent from clinical strains [61]. This could be partly due to the hydrophobic barrier that PDIMs form, which though useful in the hostile environment of the host, also prevents the diffusion of useful solutes. PE/PPE proteins, a family named for their N-terminal repeated regions of proline-(proline)-glutamate, have recently been shown to compensate for this, forming pores that selectively uptake the necessary nutrients [58].

PGLs are less prevalent in *Mtb* isolates [62,63], though are present in the *Mtb* Canetti strain [63], *M. leprae* [64], *M. marinum* [65] and *M. bovis* [66], and could be responsible for the hyper-virulence and drug resistance of the W-Beijing family of *Mtb* [8,10]. PGLs affect virulence by suppressing the release of cytokines involved in the inflammatory response of the immune system [8]. In *M. leprae*, PGLs are also implicated in the Schwann cell invasion [67] and nerve demyelination [68]. The presence of PGLs is directed by a single fused polyketide synthase (PKS), Pks15/1, which is knocked out in strains devoid of PGLs by an insertion that splits the *pks15/1* gene in two [62]. PKS proteins are required to condense, extend and modify nascent fatty acids; this particular PKS, Pks15/1, is proposed to extend from the phenol group, producing an intermediate in the synthesis of the phenolphthiocerol moiety, *p*-hydroxyphenylalkanoate (PHPA) [69]. This is extended by the PpsABCDE PKS proteins to phenolphthiocerol [70]. Mycocerosic acid synthase (MAS), a PKS located in the same cluster as the *pps* operon, produces the methyl-branched mycocerosic acids [71]. The latter two PKS systems are responsible for the synthesis of the lipid core common to PDIMs and PGLs.

Acyl trehaloses, trehalose glycolipids (SGLs) acylated with multimethyl branched fatty acids, are present in the MOM of all virulent strains of mycobacteria [49,72–74]. As such, they are important in virulence with a role in blocking phagosome maturation [75] and suppressing the host's immune system by inhibiting T cell proliferation and down-regulating cytokine secretion in activated monocytes [76–78]. The core structure consists of a central trehalose moiety that is esterified on the 2-position with a saturated fatty acid (C_16_ or C_18_); the trehalose is additionally esterified with as many as four multimethyl branched long-chain fatty acids. The simplest forms are the diacyl trehaloses (DATs) (Figure 3c), which are based on mycosanoic (C_24_) and mycolipanolic (C_27_) acids [79]. Pentaacyl trehaloses (PATs) (Figure 3d) contain mycolipenates (C_27_), whereas sulphated SGLs are made up of the longer fatty acids, phthioceranates (C_37_) and hydroxyphthioceranates (C_40_), and the trehalose moiety is sulphated at the 2′-position (Figure 3e) [80,81].

Lipooligosaccharides (LOS) are extremely polar lipids, which are associated with biofilm formation and motility [82]. They consist of a common trehalose-containing saccharide core, acylated on the trehalose moiety with multimethyl branched fatty acids (Figure 3f). The trehalose is further glycosylated with a mono-, or more commonly, an oligosaccharide of 2–6 sugars, which is species-specific [83,84]. LOS are lacking in many *Mtb* strains, though are present in *Mtb* Canetti strain [85], *M. kansasii* [86], *M. gordonae*, *M. smegmatis* [87], *M. marinum* [88,89] and a member of the *M. fortuitum* complex [90,91], later classified as *M. houstonense.* Those strains that do produce LOS are characterised by a smooth appearance, whereas those lacking these glycolipids are rough and have increased hydrophobicity over the outer membrane [92,93]. LOS synthesis requires the presence of two proximal *pks5* gene homologues flanking an associated *pap* (PKS associated protein) gene. Strains lacking LOS expression appear to have undergone a recombination-deletion event between the two *pks5* homologues, losing a *pks5-pap* segment [94]. Studies have demonstrated that LOS reduces virulence; smooth *M. kanasii* was unable to establish the chronic infection in the lungs of mice that their rough counterparts could [92]. This could be due to the observed interference by LOS of host-pathogen interactions, perhaps by covering up critical glycolipids, and it has been speculated that this has driven their loss in virulent strains [94]. The associated hydrophobicity detected in mycobacteria lacking LOS may also have the added benefit to the pathogen of enhancing aerosol transmission [93].

Glycopeptidolipids (GPLs), present in the outer leaflet of non-tuberculosis causing mycobacteria, consist of a lipopeptidyl core, glycosylated on the peptide unit. They are important in biofilm formation, aggregation, motility, cell wall integrity and interactions with the host's immune system [95–98]. Interestingly, strains producing smaller amounts of GPL are more invasive, denoting a role in pathogenicity [99–101]. C-type GPLs (Figure 3g), which form a sub-group that are alkaline-stabile, are found in *M. smegmatis*, *M. avium*, *M. chelonae* and *M. abscessus* [91,102]; their general structure consists of a core tripeptide-amino alcohol (d-Phe-d-*allo*-Thr-D-Ala-l-alaninol), esterified with a long fatty acyl chain (C_28_) [103]. The peptide is glycosylated with a 6-deoxytalose on the *allo*-threonine and a rhamnose unit linked to the alaninol. This basic core can be further acylated, methylated and glycosylated, depending on the strain, producing different serotypes [95,104,105]. The other group of GPLs, found in *M. xenopi*, are serine-containing and alkaline-labile, consisting of a tetrapeptide core with two serines (l-Ser-l-Ser-l-Phe-d-*allo*-Thr), esterified with a simpler fatty acyl chain (C_12_) [106,107] (Figure 3h). The N-terminal serine residue is methylated, acting as the primary glycosylation site for a 6-deoxytalose unit; a di/tetrasaccharide of rhamnose (terminating in a glucose for the tetrasaccharide) is attached to the *allo*-threonine [106,107].

## Fatty acid and mycolic acid biosynthesis

**Figure 4.**
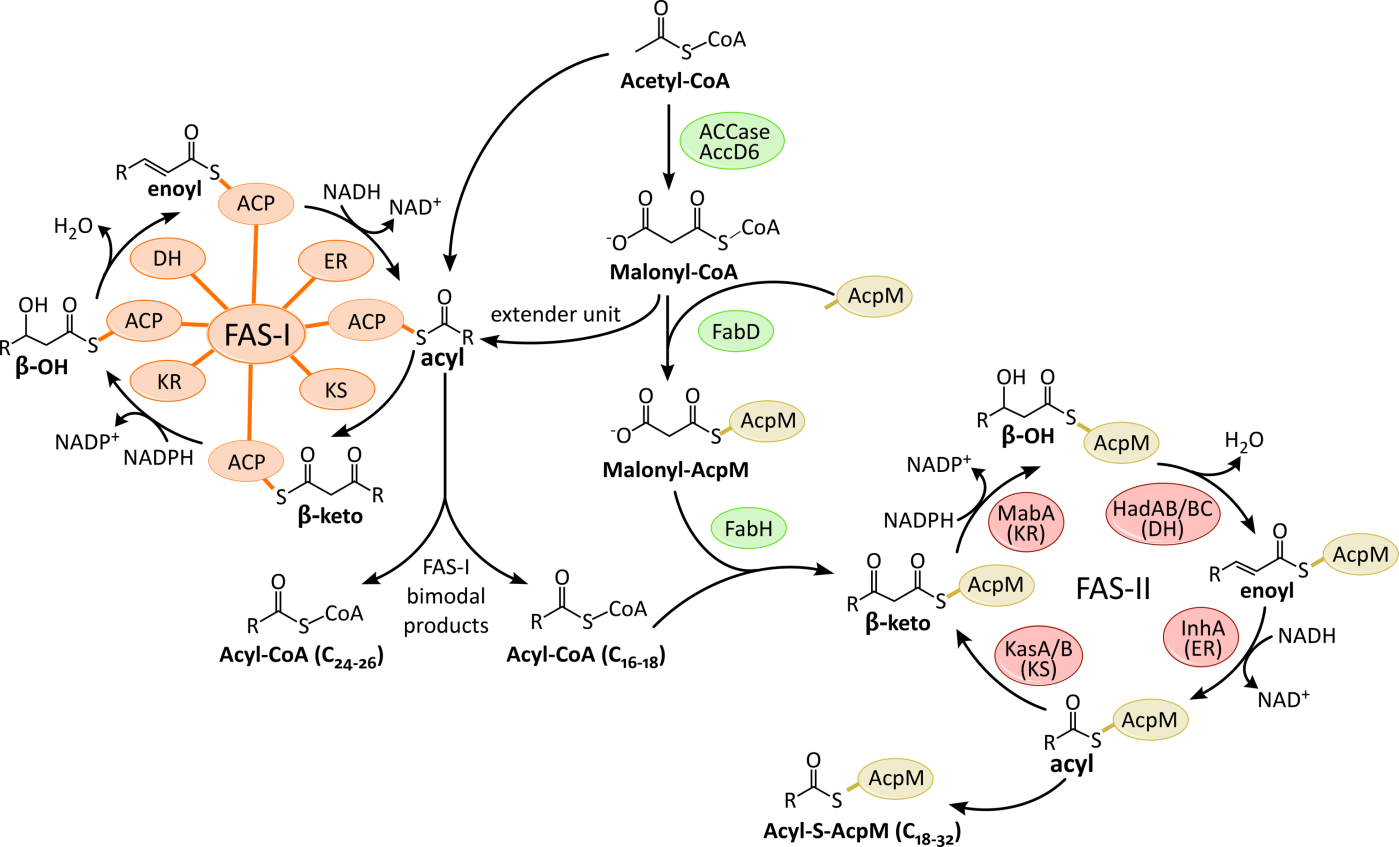
Biosynthesis of Fatty acids. For example in the production of mycolic acids (MAs). The FAS-I domains/FAS-II enzymes: KS (β-ketoacyl synthase), KR (β-keto reductase), DH (dehydratase), ER (enoyl reductase) and ACP (acyl carrier protein) [287,288].

The biosynthesis of fatty acids and MAs is carried out by two fatty acid synthases, FAS-I and FAS-II (Figure 4). FAS-I is a multi-domain ‘eukaryotic-like’ fatty acid synthase, a single large protein comprising all the catalytic domains required for fatty acid synthesis. Whereas FAS-II is a ‘prokaryotic-like’ fatty acid synthase, a large complex of discrete enzymes. FAS-I is the only system capable of synthesising fatty acids *de novo* and uniquely to mycobacteria, the synthesis is bimodal, generating fatty acids with two different chain lengths: C_24_–C_26_ (corresponds to the α-branch in MA synthesis) and C_16_–C_18_ (extended by FAS-II to produce the meromycolate chain in MA synthesis) [108–110]. These fatty acids are either shuttled to FAS-II for MA production or to other PKS systems for the further extensions and modifications required to manufacture the various complex lipids.

Both FASs extend the nascent acyl chain through sequential additions of acetate (2 carbons) from the extender unit malonyl-Coenzyme A (CoA), though FAS-II requires the malonate to be presented on an acyl carrier protein (AcpM; Rv2244) [111]. The first step in fatty acid synthesis is the production of this unit through the carboxylation of acetyl-CoA, catalysed by the acyl-CoA carboxylase complex (ACCase) [112]. FabD (Rv2243), a transacylase, next generates the malonyl-AcpM extender unit required by FAS-II, transferring the malonyl group from the phosphopantetheine group of CoA to the same prosthetic group present on the active site serine of the AcpM [113].

The FAS-I gene present in *Mtb* (*fas*; Rv2524c) contains seven domains for fatty acid synthesis: acyl transferase (AT), enoyl reductase (ER), dehydratase (DH), malonyl/palmitoyl transferase (MPT), acyl carrier protein (ACP), β-keto reductase (KR) and β-ketoacyl synthase (KS) [114]. Synthesis is initiated with an acetyl group, which is transferred from the CoA to the ACP domain. During synthesis, the growing acyl chain remains bound to this domain.

In the FAS-II system, the discrete enzymes have corresponding functions to the domains of FAS-I. The first step of synthesis in this system is an important link between FAS-I and FAS-II. FabH (Rv0533c), a β-ketoacyl ACP synthase, plays a pivotal role in initiating FAS-II synthesis through the Claisen condensation of the shorter product from FAS-I (acyl-CoA, C_16–18_) with a malonyl-AcpM [115]. This forms a β-ketoacyl-AcpM, which can then be presented to the FAS-II enzymes, commencing a round of modifications: reduction in the keto group by the keto reductase (MabA; Rv1483), dehydration of the β-hydroxylacyl-AcpM intermediate by the dehydratase heterodimers (HadAB and HadBC; Rv0635–Rv0636 and Rv0636–Rv0637), and finally reduction in the enoyl-AcpM to acyl-AcpM by the enoyl reductase (InhA; Rv1484) [30,116–118]. After the first cycle, further rounds are initiated by KasA/B (Rv2245 and Rv2246), ketosynthases which then elongate the acyl chain by the addition of two carbons each cycle, forming a β-ketoacyl-AcpM intermediate [119,120]. The completed Acyl-AcpM undergoes further rounds of elongations, desaturations and also modifications, including *cis*-/*trans*-cyclopropanation and methoxy/keto group additions [31,34,121,122].

How the FAS systems regulate the length of the growing acyl chains is not clear. Though not essential, mutations in *kasB* result in MAs shorter by 2–4 carbons, suggesting that KasB is required for full-length extensions [120,123]. The HadBC heterodimer is also associated with later elongation cycles and is similarly expected to play a role in regulating the acyl chain length in FAS-II [117,124]. The mechanism by which FAS-I is able to synthesise two products with different chain lengths (C_16_–C_18_ and C_24_–C_26_) is likely to be a complex interplay between products and enzymes. Replacing the *M. smegmatis* FAS-I system with the *Mtb* homologue, did not alter the length of the longer fatty acid chains produced in *M. smegmatis* from the usual C_24_ to the C_26_ produced by *Mtb*; this indicates that regulation is not restricted to FAS-I and may involve interactions between FabH and FAS-II with FAS-I [125]. Endogenous polysaccharides also affect FAS-I product formation, favouring the synthesis of shorter chain fatty acids (C_16_–C_18_) as well as increasing the overall rate of synthesis; these polysaccharides, containing 3-*O*-methyl-d-mannose or 6-*O*-methyl-d-glucose, are proposed to enhance product release, a rate-limiting step in the synthesis, by forming a complex specifically with the shorter length product and thereby facilitating its release [126–128]. In the absence of these polysaccharides, the synthesis of the longer fatty acid chain is favoured (C_24_–C_26_), which as longer, shows an increased ability to self-interact, forming stable aggregates that also enable product release from FAS-I [126].

## Polyketide synthases (PKS)

**Figure 5.**
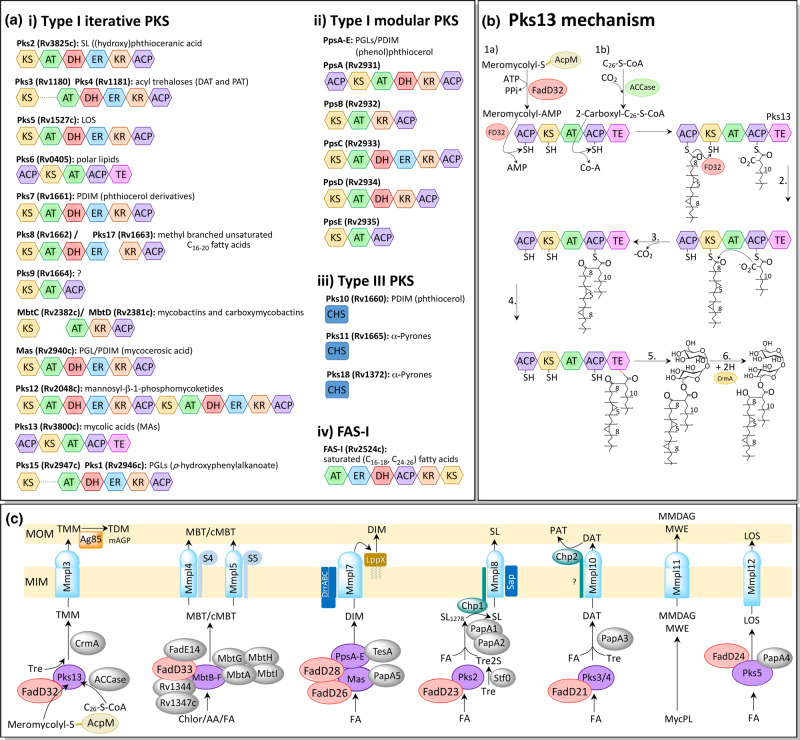
Polyketide synthase (PKS)/FAS-I domains and synthesis pathways. (**a**) Domains. The PKS domains are: AT (acyl transferase; green), ER (enoyl reductase; blue), DH (dehydratase; red), ACP (acyl carrier protein; purple), KR (β-keto reductase; brown), KS (β-ketoacyl synthase; yellow), TE (thioesterase; pink) and CHS (chalcone synthase; dark blue). Other abbreviations: SL = sulfolipids, DAT = diacyl trehaloses, PAT = pentaacyl trehaloses, LOS = lipooligosaccharides, PGL = phenolic glycolipids, PDIM = phthiocerol dimycocerosate [289–291]. (**b**) Suggested mechanism of Claisen-type condensation by Pks13. **1.a** FadD32 (FD32) activates the meromycolyl-S-AcpM with AMP and loads it on the N-terminal ACP (acyl carrier protein) domain of Pks13. **1.b** The acyl-CoA (C_26_) produced by FAS-I is carboxylated by ACCase and loaded onto the C-terminal ACP domain of Pks13 by the AT (acyltransferase) domain. **2.** The meromycolyl chain is transferred, in the presence of FD32, to the KS (ketosynthase) domain of PKS13. **3.** The Claisen-type condensation reaction: the acidic α-carbon of the carboxyl-acyl group attacks the carbonyl group of the meromycolyl chain, producing 3-oxo-C_78_-α-mycolate, releasing CO_2_ and the meromycolyl chain from the KS domain. **4.** The TE (thioesterase) domain cleaves the product from the ACP domain, possibly forming a transient covalent bond via an active site serine. **5.** The TE domain transfers the product onto a trehalose unit. **6.** The keto group is reduced by CmrA, producing the mature trehalose monomycolate (TMM) [138]. (**c**) MmpL transport of complex lipids across the cytoplasmic membrane in *Mtb*. PKS (polyketide synthase) proteins are purple, FadD are red and MmpL/S blue. S4 and S5 are MmpS4 and MmpS5, respectively; MmpL4 and 5 are involved in iron scavenging, exporting two siderophores, lipophilic mycobactin (MBT) and hydrophilic carboxymycobactin (cMBT). MmpL11 exports monomero-mycolyl diacylglycerol (MMDAG) and mycolate wax ester (MWE) from the intermediate mycolyl phospholipid (MycPL). Other abbreviations include FA = fatty acid, Tre = trehalose, mAGP = mycoloylarabinogalactan-peptidoglycan [292–294].

The mycobacterial genome boasts a large number of PKS, fundamental in the synthesis of the cell envelope's complex lipids, performing condensations, extensions and modifications of fatty acid chains (Figure 5a) [129]. There are three types of PKS systems, with distinct organisations and mechanisms. Multifunctional type I PKSs are large polypeptides with several catalytic domains. Pks12, for example, is the largest encoded polypeptide in *Mtb*, at 4151 amino acids [129]. There are two varieties of type I PKSs: iterative systems cycle the product several times through the same domains in a mechanism resembling FAS-I, whereas modular PKSs possess a single domain for each catalytic step of the entire synthesis. Type II PKSs are similar to FAS-II, comprising a large complex of enzymes, each responsible for a catalytic step. There are also three type III plant-like PKSs present in the genome of *Mtb* [129], condensing enzymes from the chalcone synthase (CHS) superfamily [130]. These enzymes are distinct from the other PKS classes, both in their structure, which is typically a homodimer of KS domains, and mechanism, acting on free acyl-CoA thioesters with no ACP involvement [131].

PKSs do not carry out *de novo* fatty acid synthesis, instead using fatty acid precursors from FAS-I. These are usually activated and loaded onto the ACP domain of the PKS by an associated FadD protein. The attached precursor is then modified by the catalytic domains of the PKS, cycling through successive extensions by the KS (β-ketoacyl synthase) domain, usually from a (methyl)malonyl-CoA extender unit via a Claisen-type condensation reaction; reduction in the keto group by the KR (β-keto reductase) domain; dehydration by the DH (dehydratase) domain and reduction in the enoyl group by the ER (enoyl reductase) domain. Some more complex lipids in mycobacteria require several PKSs to synthesise the different lipid components. For instance, in PGL synthesis, Pks15/1 synthesises the PHPA intermediate [62], PpsABCDE extends this to phenolphthiocerol [70] and mycocerosic acid synthase (MAS), produces the methyl-branched mycocerosic acids that are attached to the diols [71].

Pks13, an iterative type I PKS, is involved in the synthesis of MAs, joining together the acyl-CoA (C_24–26_) product of FAS-I with the meromycolyl-AcpM product from FAS-II, via a Claisen-type condensation reaction (Figure 5b). In this process, both substrates are first attached covalently as thioesters to the ACP domains of Pks13. The meromycolyl-AcpM is activated by FadD32 (Rv3801c) to an AMP derivative and loaded on to the N-terminal ACP of Pks13 [132,133]; the acyl-CoA fatty acid is carboxylated, by an ACCase [134,135], and loaded onto the C-terminal ACP of Pks13, via the AT domain [136]. The meromycolyl group is then transferred, in the presence of FadD32 [136], to the KS domain of Pks13 [132]. The carboxylation step is essential for the Claisen-type reaction that follows, activating the α-carbon and enabling a decarboxylative condensation reaction [137]. This proceeds via a nucleophilic attack by the acidic α-carbon of the carboxyl-acyl group towards the carbonyl group of the meromycolyl chain, releasing CO_2_ and the meromycolyl chain from the KS domain [138]. The thioesterase (TE) domain of Pks13 next cleaves the α-alkyl β-ketoacyl product from the ACP domain, possibly forming a transient covalent bond with the product via a serine in the active site; the TE domain then catalyses the transfer of the acyl chain onto a trehalose, forming α-alkyl β-ketoacyl trehalose monomycolate (*TMMk*) [139]. The final mature TMM is formed when the keto group is reduced by CmrA (Rv2509) [140,141].

Trehalose is a significant sugar in mycobacterial lipid production (for review see [142]); in addition to its role in MA synthesis, trehalose constitutes the core of the polyacyl-trehaloses, sulfolipids and LOS [138,143–147]. A disaccharide of α(1 → 1)-linked glucose, trehalose is well-known to protect against the stresses of heat, freezing, desiccation and γ-radiation [148–150]. During MA synthesis, the trehalose could be acting to shield the extreme hydrophobicity of the very long fatty acid chains, enabling export and attachment to the AG. For this reason, it would seem unlikely that the free mycolates found in the MOM, particularly during biofilm formation [42], are released from Pks13 as free mycolates, but perhaps produced and transported as TMM, and the trehalose removed by enzymatic hydrolysis in the outer membrane. After the MA is attached to the AG by the mycolyltransferases of the antigen 85 complex (Ag85) [151], the trehalose is recycled back into the cytoplasm by the ABC sugar transporter, LpqY-SugA-SugB-SugC (Rv1235–Rv1236–Rv1237–Rv1238); this transporter is essential for virulence and again demonstrates the importance of trehalose to the bacterium [152].

## MmpL/S

MmpL (mycobacterial membrane proteins large) proteins belong to the RND (resistance, nodulation and cell division) superfamily of membrane proteins, which are typically characterised by 12-transmembrane (TM) helices and two large substrate-specific periplasmic loops between TM1–2 and TM7–8 [153,154]. The energy for their activity is derived from a proton gradient across the inner membrane [155] and they usually function as antiporters, coupling the energy from proton movement into the cytoplasm, with substrate efflux [156,157]. The *Mtb* genome encodes 13 MmpL proteins, many of which are found in the same loci as PKS proteins and other proteins involved in lipid synthesis [129,158]. Though MmpL5 and MmpL7 have been shown to have roles in drug resistance and efflux [159–161], the primary function of the MmpL proteins is to export the complex lipids synthesised in the cytoplasm across the inner membrane (Figure 5c). Some MmpL proteins (1, 2, 4 and 5) have smaller, transcriptionally coupled accessory proteins, MmpS (mycobacterial membrane proteins small) [129]. Other MmpL proteins also require additional membrane proteins; MmpL8 interacts with the membrane protein, Sap [162] and transport by MmpL7 involves the ABC transporter, DrrABC, and lipoprotein, LppX [57,163].

Knock-out studies have shown that MmpL3 (Rv0206c) is the only essential MmpL protein, though others are required for virulence [158]. MmpL3 is responsible for transporting the MA precursor, TMM, across the inner membrane. Inhibitors that lead to TMM accumulation in the cytoplasm and simultaneous loss of TDM in the periplasm also selected for spontaneous resistance mutations in *mmpL3* [164,165], findings that have since been confirmed by a *mmpL3* conditional mutant in *M. smegmatis* [164,166]. Several inhibitors of MmpL3 have recently been found though spontaneous resistance based screening [164,165,167], though some of these are broad-spectrum, targeting bacterial and fungal species lacking MAs, leading to speculation that the real target could be elsewhere. The crystal structure of the homologue from *M. smegmatis*, however, has demonstrated that many of these inhibitors, including SQ109, do bind directly to MmpL3 [168]. The resolved structure consists of a central proton translocating channel, formed between 2 TM helices (TM4 and TM10) that intertwine and are linked by hydrogen-bonding between two sets of Asp-Tyr residues. These interactions, which are essential for proton relay, were disrupted by binding of the inhibitors, all of which co-crystallised in the central pore [168]. While many RND transporters form homo-dimers or -trimers [168,169–171], and a homology model refined with electron microscopy (EM) suggested MmpL3 is a trimer [172], in the crystal structure MmpL3 was monomeric [168].

MmpL protein expression is controlled by transcriptional repressors, which are in turn regulated by the availability of fatty acids; MmpL3 is regulated by three TetR regulators: Rv3249c, Rv0302 and Rv1816, all of which dissociate from DNA upon binding palmitic acid, relieving transcriptional repression [173–175]. Phosphorylation of the large C-terminal domain possessed by MmpL3 has also been implicated as a post-translational regulation system [176].

## Phosphatidyl-*myo*-inositol mannosides (PIMs), lipomannan (LM) and lipoarabinomannan (LAM)

**Figure 6.**
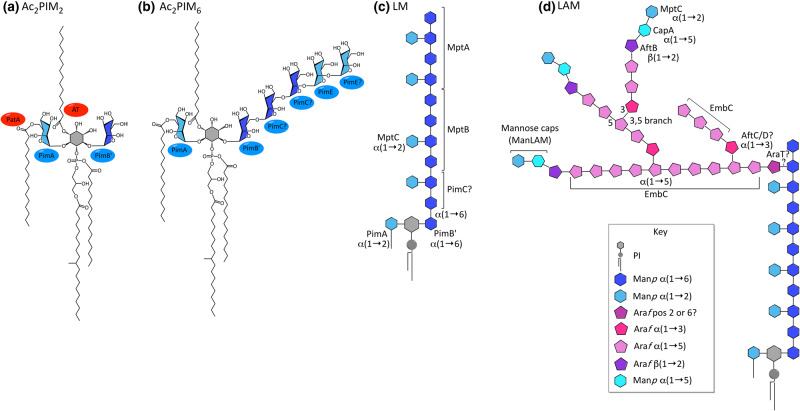
Structure of phosphatidyl-*myo*-inositol mannosides, Lipomannan and Lipoarabinomannan. (**a**) Ac_2_PIM_2_, (**b**) Ac_2_PIM_6_, (**c**) LM and (**d**) LAM (and ManLAM). See key for component sugars [23].

These lipoglycans (Figure 6) comprise a phosphatidyl-*myo*-inositol (PI) core decorated with mannose residues (Man*p*, pyranose ring form), with additional branches of arabinose (Ara*f*, furanose ring) in LAM. Acyl chains on the inositol, glycerol and mannose moieties provide lipid anchors into the inner and possibly also the outer membranes [25,74,177], though the inner membrane is perhaps their principal location as LM and LAM possess the same lipid anchors as the PIMs, which share this location [22,23]. They are ubiquitous in mycobacteria, with important roles in stability, permeability and cell division [178–180]. LM and LAM are also important in virulence, modulating interactions with the immune system [7,9]; LAM, in particular, plays a key role in the infection life cycle of *Mtb*, blocking phagosome maturation and therefore avoiding lysis and antigen presentation, enabling *Mtb* to avoid the host's defences whilst providing a habitable environment in which to replicate [181]. Synthesis proceeds via PIM → LM → LAM [182], yet PIM_2_ and PIM_6_ are not only intermediates in the synthesis of LM and LAM, but end products in their own right, forming much of the cytoplasmic membrane, enhancing the stability and reducing drug permeability [22]. In fact, PIM_6_ is unlikely to be intermediary, possessing two α(1 → 2) linked mannose residues not present in LM and LAM; instead PIM_4_ is the likely branch point between the two pathways [183]. Structurally, in PIM_2_ (Figure 6a), the 2 and 6 positions of the *myo*-inositol ring each have a single linked mannose residue, whereas in PIM_6_ (Figure 6b), a chain of five mannose units resides at the 6-position in addition to the mannose at position 2 [184]. Both LM and LAM possess a core chain of 21–34 α(1 →6) linked mannose residues from position 6 of the *myo*-inositol ring, dotted with single α(1 → 2) linked mannose units (Figure 6c) [185]. LAM has an additional arabinan domain of highly branched arabinose residues, similar to AG (Figure 6d) [186]. The non-reducing termini of these arabinose branches can be capped mannose residues to form ManLAM [187].

The biosynthesis of PIMs, LM and LAM is compartmentalised, which is achieved by the presence of both soluble, membrane-associated glycosyltransferases (GTs) in the cytoplasm (from the GT-A/B superfamily) and membrane-bound GTs (from the GT-C superfamily) in the periplasm [188,189]. This enables the initial glycosylation of the lower PIMs to occur within the cell; the lipid-anchored PI sits on the cytoplasmic face of the inner membrane, with the inositol moiety pointing inwards. Here, mannose is transferred from a GDP-Mannose sugar donor, initially with α(1 → 2) linkage to position 2 of the *myo*-inositol by PimA (Rv2610c), forming PIM_1_ [190,191]; and then with α(1 → 6) linkage to position 6 by PimB’ (Rv2188c), forming PIM_2_ (Figure 6a) [192,193]. An acyl chain (palmitate) is transferred by PatA (Rv2611c) to position 6 of the mannose added by PimA, forming Ac_1_PIM_2_, which has three acyl chains [194,195]; position 3 of the *myo*-inositol ring can also be acylated by an unknown acyl transferase to Ac_2_PIM_2_, a tetra-acylated form, though LM and LAM are predominantly triacylated [183]. PIM_2_ either remains in the inner leaflet of the cytoplasm where it forms the majority of the lipid content [22], or is further mannosylated on position 6 with α(1 → 6) linkages to PIM_4_. The GT(s) responsible for this has yet to be identified; PimC was found in the genome of *Mtb* CDC1551 as a possible candidate for the mannosylation to PIM_3_, though there is no homologue in some strains of mycobacteria (*Mtb* H37Rv and *M. smegmatis*) and a knock-out in *M. bovis* did not reduce the levels of PIMs, LM or LAM, indicating that another pathway must exist [196].

The biosynthesis of the higher PIMs, LM and LAM occurs outside of the cytoplasm, though it is not known whether the juncture for this is PIM_2_, PIM_3_ or PIM_4_ [197]. The PIM is flipped across the inner membrane so that the inositol and mannose moieties now point into the periplasm. A candidate for this transport is the putative ABC transporter, Rv1747, a knock-out of which had low levels of PIMs and showed reduced virulence [198]. However, this mutant still had WT levels of LM and LAM, suggesting the presence of an additional transport mechanism [199]. In the periplasm, membrane-bound GTs transfer further mannose units from the lipid-linked donor, polyprenyl-mannose [182]. The formation of PIM_6_ from PIM_4_ (Figure 6b) involves the addition of two α(1 →2) linked mannose residues, the first or both of which are transferred by PimE (Rv1159) [200]. In LM and LAM biosynthesis, the α(1 → 6) linked mannose core is extended from PIM_4_ by MptB (Rv1459c) [201], followed by MptA (Rv2174), an order determined by knock-outs. The deletion of MptA in *C. glutamicum* resulted in a truncated LM and halted LAM production, which was complemented by Rv2174 or the *C. glutamicum* homologue [202]. The deletion of MptB in *C. glutamicum* completely abolished all LM and LAM synthesis, which was also successfully complemented [201]. A similar knock-out of MptB in *M. smegmatis*, however, did not affect LM/LAM production, allowing for some redundancy in this pathway [201]. The α(1 → 2) linked mannose residues that decorate the mannose core, producing mature LM, are added by MptC (Rv2181) [203,204], a knock-out of which abolished all LM synthesis in *M. smegmatis*, though still produced a truncated version of LAM; the immature LM is speculated to be either degraded or processed into LAM [201]. The regulation of the branch point of PIM_4_ to either PIM_6_ or LM is controlled by LpqW (Rv1166). Disruption of the corresponding gene in *M. smegmatis* reduced LM/LAM synthesis, producing an unstable mutant that restored LM/LAM levels through a spontaneous mutation disrupting *pimE*, which simultaneously abolished the dependent PIM_6_ pathway [205,206]. A similar deletion in *Corynebacterium glutamicum*, which lacks a PimE homologue and higher PIMs, was stabilised by a point mutation in *mptB*. MptB, which is required for the initial mannosylation of the LM core, was subsequently shown to have reduced activity in the absence of LpqW and authors speculate that this mechanism could regulate MptB and subsequently LM synthesis [207]. GDP could also be a regulator, it has been shown to inhibit early PIM synthesis and reduce levels of the polyprenyl-mannose substrate, perhaps by reversing the synthesis of this substrate to GDP-Mannose [197].

The arabinan domain of LAM is similar to that of arabinoagalactan (AG), sharing the same linkages and branched core of 18–22 arabinose units [208], though LAM is more variable in structure [208–211] and is characterised by the presence of more linear chains at the reducing end. AG is terminated by a distinctive branched hexa-arabinoside unit (β-d-Ara*f*(1 → 2)α-d-Ara*f*]_2_-3,5-α-d-Ara*f*-(1 → 5)-α-d-Ara*f*) [212], whereas LAM also has the linear tetra-arabinoside motif (Ara*f*_4_; a chain of four α(1 → 5) linked Ara*f*) [186]. This is unsurprising since both domains are constructed by a similar set of membrane-bound arabinosyltransferases, using the lipid-linked donor, decaprenylphosphoryl-d-arabinose (DPA) [213]. During synthesis, the mannan core of the LM is first primed, though the arabinosyltransferase responsible and the location of this attachment are not known; while the *O*-2 of the mannose was originally implicated [214], more recent studies suggest that the linkage is to the *O*-6 position [215], which would restrict the attachment of the arabinan domain to the very end of the mannan core. Clarification requires further analysis with longer chain saccharides. After the initial priming, EmbC (Rv3793) adds an arabinan core of 12–15 arabinose residues with α(1 → 5) linkage [208,216,217]. AftC (Rv2673), which has α(1 → 3) activity, introduces 3,5-arabinose branches in both LAM [217] and AG synthesis [218]. AftD (Rv0236c) has been shown to have similar activity to AftC and may also introduce branch points [219], though α(1 → 5) activity in AG synthesis has also been demonstrated [220]. EmbC further extends these branch points by up to 8–9 arabinose units with α(1 → 5) linkage [208–216]. Finally, AftB (Rv3805c) adds the terminal arabinose residue, with β(1 → 2) linkage, to the non-reducing ends of the arabinan [221].

Another difference in the arabinan domains of LAM and AG is the nature of the reducing end. In AG, most are esterified with MAs [5,36], while LAM is capped with mannose, producing ManLAM. This mannose capping is species-specific: in *Mtb* and other slow-growing virulent strains, the LAM is capped with up to three linear α(1 → 2) linked mannose residues (Man*p*) on the 5-position of the terminal arabinose [187]; in faster-growing mycobacteria, such as *M. smegmatis*, much of the LAM is uncapped, while a portion is capped with phosphoinositide (PILAM) [222]; *M. leprae* has much reduced mannose capping and *M. chelonae* has none at all [222,223]. Capping is initiated by CapA (Rv1635c), which adds the first mannose residue with α(1 → 5) linkage [224,225]. Further residues are next attached by MptC, the mannosyltransferase responsible for adding the α(1 → 2) mannose to the mannan core [226].

Cell surface labelling, using either biotin followed by lipid extraction or antibodies and cryo-electron microscopy, has shown the presence of arabinan mannan (AM; non-acylated LAM), and also PIMs, LM and LAM, on the surface of the mycobacterial envelope; the PIMs and LM/LAM have lipid anchors and are thought to sit within the lipids of the MOM, projecting into the capsule, while the AM lacks this anchor and is free within the capsular layer [25,74,177]. However, this issue is debateable and not always reproducible; a study using atomic force microscopy with immunogold labelling, for example, was only able to demonstrate the presence of surface-exposed LAM after treatment with drugs that target the cell wall [227]. Also, if PIMs, LM and LAM do indeed locate to the MOM, a yet undiscovered transport system would be required to extract these lipoglycans from their inner membrane anchoring and then relocate them across the periplasmic space and through the lipids of the MOM, a system hypothesised to be similar to the one used by *Escherichia coli* in the transport of LPS [177,228]. The presence of PIMs, LM and LAM in the MOM would certainly be reassuring, since surface exposure is essential for their observed roles in infection and interactions with the host's immune system, especially since the PIMs, LM and LAM located in the inner membrane were proven not to be surface-exposed [177]. Studies with purified ManLAM and PILAM demonstrated that the mannose cap blocks the fusion of the phagosome and expression of pro-inflammatory cytokines [181,229–231], though these results were not replicated with live bacteria deleted of *capA* and lacking mannose caps [225]. Similar mutants did, however, demonstrate that ManLAM is recognised by Dectin-2, initiating the expression of pro-inflammatory cytokines [232]. LM has also been shown to activate the Toll-like receptor 2 (TLR2), triggering the innate immune system. This response is proportional to the chain length of the mannan core but is reduced with LAM, possibly because the mannan core is obscured by the arabinan domain [233–236]; truncations of the arabinan domain of LAM restored this activation [217,221]. While initial stimulation of the TLR2 activates the immune response, prolonged stimulation in macrophages has been shown to have the opposite effect, preventing antigen presentation by major histocompatibility complex class II molecules (MHC) [237,238]. This could be a crucial part of the mechanism employed by *Mtb* to evade the host's immune system and establish a latent phase infection in macrophages [239].

## Mycobacterial antigens and CD1 presentation

For many years, it had been assumed that proteins are the sole antigens, activating T cells via the T cell receptor (TCR), and lipids were limited to stimulating the innate immune system. More recently, it has been recognised that many mycobacterial lipids, including mycolates [240], PIM [241–243], LAM [241] and phosphatidylglycerol (PG) [244], activate T cells through the CD1 (cluster of differentiation molecules) presentation pathway (Table 1). CD1 proteins are similar to MHC molecules [245], which present peptide antigens on antigen-presenting cells (APCs) to conventional T cells via the TCR, though CD1 presentation is limited to CD1-restricted T cells. There are five human CD1 proteins: CD1a, -b, -c and -d are membrane proteins involved in presenting lipids and are expressed on APCs, such as B cells, Langerhans cells and dendritic cells as well as cortical thymocytes [246]. CD1e is a soluble protein that processes the glycolipids in order to increase the versatility of the CD1 proteins. CD1e functions by binding directly to the glycolipids and aiding their enzymatic digestion, for example by α-mannosidase in PIM_6_ presentation by CD1b [243].

**Table 1.** **Human CD1 and the mycobacterial lipids they present** [249]

Human CD1a	Human CD1b	Human CD1c	Human CD1d	Human CD1e
Dideoxy-mycobactin [259]	Mycolate [240]	Mannosyl mycoketide [260]	Phosphatidylglycerol [244]	PIM [243]
	Glucose monomycolate [261,262]	Phosphomycoketide [263]	Cardiolipin [244,264]	
	Glycerol monomycolate [253]		Phosphatidylinositol [244,264]	
	Sulfoglycolipid [266]	Mannosyl-β-1-phosphoisoprenoids [265]		
	PIM [243]		PIM [242]	
	LAM [241]			
	Phosphatidylglycerol [267]			

The expression of the CD1 proteins is regulated by the TLR, via interleukin-1β, a pathway that is triggered upon *Mtb* infection [247,248]. The lipid antigen binds to CD1 either directly at the cell surface or within the endosome, after the antigen or whole bacteria has been phagocytosed into the cell, a mechanism that enables additional processing by CD1e [249]. If binding occurs in the endosome, the CD1-lipid complex is then trafficked to the cell surface in order to present the antigen to the TCR of CD1-restricted T cells [249]. CD1b and CD1c are better adapted to binding to lipids within endosomal compartments [240,250], whereas CD1a is more suited to binding to antigens at the cell surface [251]. Studies have shown that there are higher levels of CD1-restricted T cells present in individuals exposed to *Mtb* [252,253] and their activation by CD1 presenting molecules is thought to function in the host defences. The T cell response to this activation is not known in humans, though *in vitro* effector mechanisms have been demonstrated to include the secretion of alpha and gamma interferon and granulysin production, which results in lysis of infected cells and macrophage activation [254–256]. CD1d is recognised by CD1d-restricted natural killer (NK) T cells, which activate both the innate and the adaptive immune system, with responses that include proliferation and cytokine secretion, including gamma interferon and interleukin-4. Invariant NKT cells are also important in granuloma formation and are recruited to the site by lipid antigens, though the evidence suggests that CD1d is not involved in their presentation [257,258].

## Conclusions

*Mtb* commands a complex array of lipids, many of which are crucial to its pathogenicity and survival, with roles in stability, drug permeability, cell division, biofilm formation, infection and host interactions. Many of the lipids present, such as the MAs and the lipid anchors of the PIMs, are packed together tightly in parallel arrangements, a feature that greatly enhances structural stability, while providing a generous hydrophobic barrier that reduces permeability to drugs. Free lipids provide yet more hydrophobic coating, constituting the outer layer of the MOM, an unconventional second membrane. The distinctiveness of the *Mtb* cell envelope does not end here as the bulk of the complex lipids present form part of much larger complexes, mAGP and LM/LAM, structures essential for viability that are unique to the *Corynebacterium* genus.

While it might be easy to consider the cell envelope to be a rigid, unchanging entity, it is in fact dynamic. For instance, the conformation of the MAs is temperature dependent. Also the lipid content of the envelope varies according to the infectivity cycle (for review see [268]). PIMs are up-regulated during early infection, while LM and LAM are more likely to predominate during latent phase [43,268]; the high levels of LAM expressed in the granulomas may guard against the host's defences, masking the mannan core [233–235]. Prolonged stimulation of the TLR2 by LM could also assist in establishing and maintaining the granulomas, by inhibiting antigen presentation in the macrophages and thus evading the host's immune response [239]. MAs, as an essential component of the cell wall, are synthesised in line with growth, though their structural composition is thought to vary throughout [268,269]. The TDM and TMM levels, however, decrease in stationary phase, accompanied by an increase in the levels of free mycolates [43], which could be an initial step in biofilm formation [42]. In addition, PDIM is highly synthesised in actively growing cells, but not in stasis [268,269], correlating with the crucial role that it plays during active infection [270].

Many of the proteins involved in lipid synthesis and transport are essential and as such represent ideal drug targets. Indeed, many the current antibiotics already inhibit some of the enzymes involved, including isoniazid (INH) and ethionamide (ETH), both of which target InhA, abolishing MA biosynthesis [271–273]; ethambutol inhibits the arabinosyltransferases involved in assembling the branched arabinan domains of both LAM and AG, blocking MA attachment to the latter [274–278]; the recently approved second-line drugs delamanid and pretomanid also affect MA synthesis, though the exact targets are not known at present [279,280]; and not to mention the plethora of compounds in the pipe-line that look to be inhibiting MA transport by MmpL3 [164,165,167]. Future research into the area would benefit by studying the MmpL proteins, key lipid transporters present in the inner membrane, one of which is essential for survival (MmpL3) and many more are required for pathogenicity. Searching for the enzymes missing from some of the biosynthesis pathways could also prove fruitful, as would structural data and *in vitro* assay development for all appropriate targets. Today much of the current drug discovery works backwards from the drug to the target; though in the future, as our knowledge of this organism grows and enzyme assays are developed, screening could become increasingly tailored to the target. The cell envelope of *Mtb* is truly unique, contributing to its triumph as the most successful pathogen of our time. An understanding of its structure and biosynthesis, therefore, is crucial in the search for new drugs to tackle the ever-growing tide of resistance.

## Open Access

Open access for this article was enabled by the participation of University of Birmingham in an all-inclusive *Read & Publish* pilot with Portland Press and the Biochemical Society under a transformative agreement with JISC.

## Abbreviations

ACP: acyl carrier protein
AG: arabinogalactan
AT: acyl transferase
DH: dehydratase
ER: enoyl reductase
GPLs: glycopeptidolipids
GTs: glycosyltransferases
KR: keto reductase
KS: ketoacyl synthase
LAM: lipoarabinomannan
LM: lipomannan
LOS: lipooligosaccharides
mAGP: mycoloylarabinogalactan–peptidoglycan complex
MAS: mycocerosic acid synthase
MPT: malonyl/palmitoyl transferase
PDIMs: phthiocerol dimycocerosate
PGLs: phenolic glycolipids
PHPA: *p*-hydroxyphenylalkanoate
PIMs: phosphatidyl-*myo*-inositol mannoside
PKS: polyketide synthase
RND: resistance, nodulation and cell division
SGLs: sulphated trehalose glycolipids
TCR: T cell receptor
TDM: trehalose dimycolates
TLR2: toll-like receptor 2
TMM: trehalose monomycolates
XDR: extremely drug resistant
